# Chemically reactive nanofluid flow past a thin moving needle with viscous dissipation, magnetic effects and hall current

**DOI:** 10.1371/journal.pone.0249264

**Published:** 2021-04-15

**Authors:** Arshad Khan, Wiyada Kumam, Imran Khan, Anwar Saeed, Taza Gul, Poom Kumam, Ishtiaq Ali

**Affiliations:** 1 College of Aeronautical Engineering, National University of Sciences and Technology (NUST), Islamabad, Pakistan; 2 Department of Mathematics and Computer Science, Program in Applied Statistics, Faculty of Science and Technology, Rajamangala University of Technology Thanyaburi, Thanyaburi, Pathumthani, Thailand; 3 Department of Mathematics and Statistics, Bacha Khan University, Charsadda, Khyber, Pakhtunkhwa, Pakistan; 4 Department of Mathematics, Abdul Wali Khan University, Mardan, Khyber, Pakhtunkhwa, Pakistan; 5 Department of Mathematics, City University of Science and Information Technology, Peshawar, KP, Pakistan; 6 Center of Excellence in Theoretical and Computational Science (TaCS-CoE), Faculty of Science, King Mongkut’s University of Technology Thonburi (KMUTT), Bangkok, Thailand; 7 Department of Medical Research, China Medical University Hospital, China Medical University, Taichung, Taiwan; 8 Department of Mathematics and Statistics, College of Science, King Faisal University, Hafouf, Al- Ahsa, Saudi Arabia; Central University of Karnataka, INDIA

## Abstract

This work addresses the ability to manage the distribution of heat transmission for fluid flow occurs upon a paraboloid thin shaped hot needle by using hybrid nanoparticles containing Copper Oxide (*CuO*) and Silver (*Ag*) with water as pure fluid. The needle is placed horizontally in nanofluid with an application of Hall current and viscous dissipation. The popular Buongiorno model has employed in the current investigation in order to explore the impact of Brownian and thermophoretic forces exerted by the fluid. The modeled equations with boundary conditions are transformed to non-dimensional form by incorporating a suitable group of similarity variables. This set of ordinary differential equations is then solved by employing homotopy analysis method (HAM). After detail study of the current work, it has established that the flow of fluid reduces with growth in magnetic effects and volume fractions of nanoparticles. Thermal characteristics increase with augmentation of Eckert number, magnetic field, volume fractions of nanoparticles, Brownian motion parameter and decline with increase in Prandtl number. Moreover, concentration of nanoparticles reduces with corresponding growth in Lewis number and thermophoresis, chemical reaction parameters while increases with growth in Brownian motion parameter.

## 1. Introduction

The modern world is an eye witness of momentous evolution in manufacturing of numerous devices and components used in engineering applications at industrial level. At industrial level, some devices are escalating their thermal characteristics that reduce heat bearing capacity of such devices with the passage of time. For maintenance of the temperature within the prescribed design limits, a number of liquids such as air, lubricants and water etc. are required. But these pure fluids are not sufficient to fulfil the industrial requirements. Hence scientists and researchers have been endeavoring to maintain the heat transfer within the prescribed design limit by employing different procedures. The suspension of nano-sized particles in a base fluid is one of such techniques. These small particles are called nanoparticles. Firstly, Choi [1] has recommended the quantity of nanoparticles in a pure fluid for enhancing the heat transfer characteristics of such fluid. Afterwards, a number of researchers have diverted their attention to discuss the heat transmission characteristics for fluid flow by employing the concept of combination of nanoparticles to pure fluids. Hayat et al. [2] have investigated variable heat flux for nanofluid at stagnation point of thin horizontal needle. In this study the flow was supported by the stretching surface where the flow and thermal characteristics were strongly dependent upon the size and shape of the needle. The authors of this work have used carbon/water nanofluid and have solved numerically the molded problem by shooting technique. It has established in this work that growth in volume fraction of nanoparticles have resulted in enhancement of flow characteristics. Waini et al. [3] have studied transfer of heat for mixed convective hybrid nanofluid over a vertically placed thin needle by using stipulated surface heat flux. The author of this work have transformed modeled equations into set of nonlinear ODEs and then have solved numerically, that set of equations by employing bvp4c in Matlab software. It has observed in this study that size of needle and volume fraction of copper nanoparticles has a great impact on physical characteristics of needle. Khan et al. [4] have discussed the heat source and sink with melting phenomenon for an unsteady Falker-Skan flow of nanofluid. In this work the analysis for stagnation point flow characteristics have also carried out by the authors. Waini et al. [5] have discussed transfer of heat over a porous needle by using copper and alumina nanoparticles using Brownian motion and thermophoretic effects upon hybrid naofluid. In this work the numerical solution has determined by employing bvp4c in Matlab software after their transformation to dimensionless form. It has also noticed in this work that the bifurcation of solutions has occurred for negative values of moving parameter i.e. when the needle has shifted towards the origin. Moreover, it has noticed in this study that the rate of heat transmission and coefficient of skin friction have also noticed to be augmented with reduction in size of the needle. Krishna et al. [6] have carried out the boundary layer analysis for a horizontally moving needle inside Sakiadis and Blasius magnetohydrodynamics nanofluid flow using thermal radiation. The authors of this investigation have solved the modeled equations numerically by using both Runge-Kutta method and shooting technique. Khan et al. [7–10] have carried out a wonderful work for thermal flow of nanofluid by taking different flow conditions and geometries. Al-Hossainy and Eid [11–13] conducted an incredible experimental work for the flow and heat transfer by using hybrid nanofluid. Eid et al. [14] have addressed three dimensional Prandtl nanofluid flow past a convective heated surface by considering the impact of thermal radiation and chemical reaction upon flow system. Alaidrous and Eid [15] have investigated thermally radiative three dimensional nanofluid flowing past a porous surface by employing the effects of Joule heating, viscous dissipation and several slip conditions. In this work, modeled problem has solved by optimal homotopy analytical method and has established that the augmenting values of porosity, radiation and sink/source parameters have declined the Nusselt number. Eid and Nafe [16] have investigated the variation of thermal conductivity and effects of heat production upon magneto hybrid nanofluid flowing past a porous surface.

The investigations for exchange of thermal flow of fluid around various objects have attracted the investigators due to its important physical applications such as wind engineering and air flow past an aircraft etc. Lee [17] has examined the boundary layer flow around a horizontally placed needle by considering incompressible viscous fluid. In this work, a numerical solution along with the asymptotic behavior has discussed. Afterwards, this work of Lee has further modified by Narain and Uberoi [18, 19] by discussing the idea for free and forced convective flow upon a vertically placed thin needle. Upreti and Kumar [20] have discussed the Magnetohydrodynamics (MHD) nanofluid flow upon a thin needle using Joule heating effects. Sulochana et al. [21] discussed 2D forced convective MHD ferrofluid flow upon a horizontally moving needle using non-uniform heat source/sink and viscous dissipation. For checking the variations in the behavior of boundary layer, the authors of this work have used two different nanofluids by taking water and methanol with nanoparticles of Ferric-Oxide (*Fe*_3_*O*_4_). Khan et al. [22] have discussed numerically the interpretation of autocatalysis chemical reaction for a nonlinearly radiative three dimensional flow of magneto-fluid using heat source and sink. Khan et al. [23] have also discussed the production of irreversibility for a cross fluid in the presence of magnetic field and viscous dissipation. In this work it has established that the Bejan number and entropy production rate have considerably affected the thermal mass flow rates. Sulochana et al. [21] have investigated numerically the boundary layer mixed convection two dimensional flow for a persistent moving needle in the MHD ferrofluid. The authors have solved the modeled problem by employing Runge-Kutta method and have established that the augmenting size of needle has reduced the flow and thermal profiles. Khan et al. [24] have also carried out an investigation for the significance of convective thermal flow of non-Newtonian nanofluid flow by employing the famous Buongiorno model.

Due to the wide range applications and importance of chemical reaction its study has remarkably increased at industrial level. These applications include manufacturing of glass, fog creation and circulation, chemical and biological processing of different equipments and processing of food etc. When some external mass is available in the fluid, then the chemical reaction takes place in the flow system. It can be of two types i.e. homogenous and heterogeneous. The former occurs consistently in case of single phase of material such as gas, solid or liquid while the latter takes place when we have two or more than two phases such as liquid and solid or gas and solid etc. Mabood et al. [25] have examined the impacts of chemical reaction upon MHD flow for nanopartcles through a permeable medium. It has established in this work that with a growth in volume fraction of nanoparticles and magnetic field there is a corresponding augmentation in mass, heat transmission and coefficient of skin friction. Ramzan et al. [26] discussed the flow of fluid over a permeable surface using the effects of chemical reaction upon flow system. In this work, it has established that the maximum variation in source/sink has resulted in higher growth of thermal characteristics. Khan et al. [27] have discussed a comparison for Casson fluid flowing upon stretched sheet with the effects of chemical reaction. It has noticed that the flow characteristic has reduced with augmentation in Hartman number in this work. Makind and Animasaun [28] have discussed the MHD bio-convection flow for nanofluid upon horizontal surface using various flow effects. The authors of this work have used Buongiorno model and has solved modeled equations numerically by employing shooting method. Hamid [29] has studied the MHD Casson nanofluid flow upon a vertically placed thin needle using chemical reaction and non-linear thermal radiation. It has shown in this work that, with increase in thickness of the needle the rate of heat transmission of nanofluid has also augmented. Eid et al. [30] discussed the Carreau nanofluid flowing upon a convective heated nonlinear stretched surface with chemically reactive species. In this work, the authors have used the famous Buongiorno model to investigate the impact of thermophoresis and Brownian motion upon flow system.

Viscous dissipation plays a dynamic role in the fluid flow problem with different geometries. Many investigations have conducted for nanofluid flows under the impact of viscous dissipation. Ghaisi and Saleh [31] have used the Buongiorno model to discuss the numerical solution for Casson fluid flow using thermal radiation and viscous dissipation and have solved the modeled equations analytically and numerically by respective use of HAM and Runge-Kutta-Fehlberg fourth-fifth order method (RKF45M). It has established in this work that, growth of thermal layer is affected by viscous dissipation and magnetic field. Raju et al. [32] have investigated the influence of Darcy-Forchheimer flow for viscoelastic fluid upon a thin needle. Mishra and Kumar [33] examined numerically the impact of viscous dissipation upon MHD nanofluid flow over a porous surface using thermal radiation. Farooq et al. [34] studied the production of entropy for hybrid nanofluid flow upon a needle usinf viscous dissipation. The authors of this work have also carried out a comparative examination for irreversibility analysis using separately a hybrid and a pure fluid. Alotaibi et al. [35] discussed numerically the MHD flow for a Casson nanofluid upon a convective heated nonlinear stretching surface by taking into account the effects of viscous dissipation and suction injection.

We observed from the above stated and other similar literature that many studies have been presented for heat transfer regarding nanofluid flow upon a needle, but very few studies are available for hybrid nanofluid flow over a needle in the presence of different effects of surrounding medium. The current work addresses the ability to manage the distribution of heat transmission for fluid flow occurs upon a thin shaped hot needle by using hybrid nanoparicles containing silver and copper oxide with water as base fluid. The needle is placed horizontally in nanofluid using of Hall current, chemical reaction and viscous dissipation. HAM is used to establish the solution for modeled problem.

## 2. Physical description and mathematical formulation of the model

In this subsection we shall introduce our problem in physical as well as in mathematical form. First the problem will be described physically with the help of schematic diagram. Then following the physical description the problem will be modeled mathematically. In this whole phenomenon some pertinent parameters will also be encountered that will be defined mathematically with physical interpretation by end of this section.

### 2.1 Physical description of the problem

**Fig 1:**
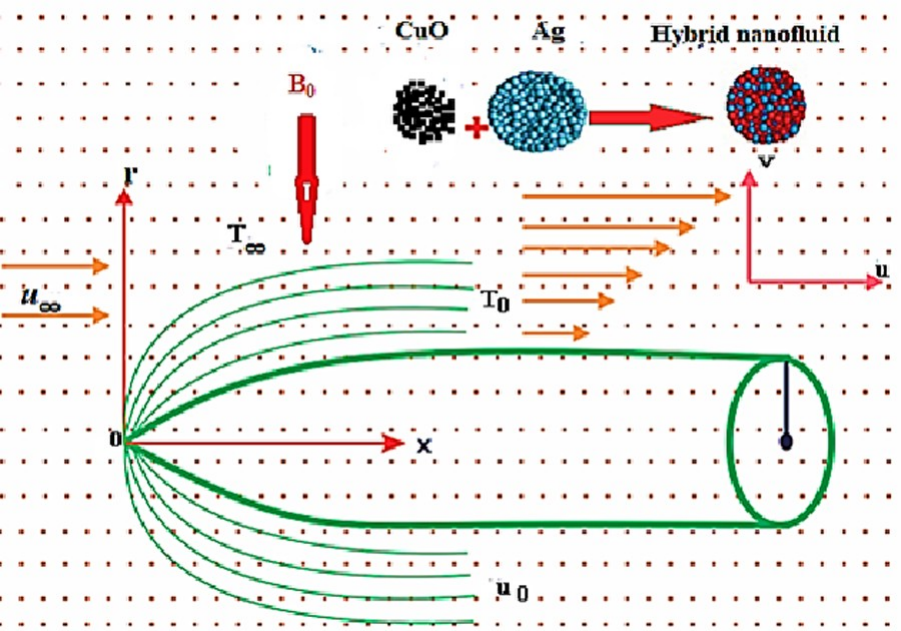
Schematic diagram of the problem.

Consider a horizontal thin heated needle surrounded by an incompressible viscous fluid and contains the nanoparticles of Silver(*Ag*) and Copper Oxide(*CuO*). Let *u*, *v* be the components of velocity along *x*(axial direction) and *r*(radial direction) respectively as shown in Fig 1. The needle is assumed to be moving horizontally with uniform velocity *u*_*w*_ in a similar or opposite direction of immersed fluid flowing above the needle with a constant velocity *u*_∞_. The radius of needle is described as *R*(*x*) = (*v*_*f*_*cx*/*U*)^1/2^ where *c* represents size of needle, *v*_*f*_ depicts kinematic viscosity while *U* = *u*_*w*_ + *u*_∞_ is the composite velocity for assumed flow system. The constant wall temperature and fluid concentration at surface of needle are *T*_*w*_, *C*_*w*_ respectively and their corresponding values for ambient fluid are *T*_∞_, *C*_∞_ such that *T*_*w*_ > *T*_∞_ and *C*_*w*_ > *C*_∞_. It is further assumed that the needle’s size is thin so that the pressure gradient is negligible while transverse curvature has certain influence.

### 2.2 Mathematical formulation

Keeping in view all the assumptions described in subsection (2.1) and applying the Buongiorno model, the governing equations of assumed flow problem transformed to [36, 37]

$$\frac{\partial \left(r\;u\right)}{\partial x}+\frac{\partial \left(r\;v\right)}{\partial r}=0$$ (1)

$$u\;\frac{\partial \;u}{\partial x}+v\;\frac{\partial u}{\partial r}=\frac{\mu_{hnf}}{\rho_{hnf}}\frac{1}{r}\frac{\partial }{\partial r}\left(r\frac{\partial u}{\partial r}\right)-\frac{\sigma B_{0}{}^{2}}{\rho_{hnf}\left(1+m^{2}\right)}u$$ (2)

$$\begin{array}{l} {\left(\rho C_{p}\right)}_{hnf}\left(u\;\frac{\partial T}{\partial x}+v\;\frac{\partial T}{\partial r}\right)=\tau \left(D_{B}\frac{\partial T}{\partial r}\frac{\partial C}{\partial r}+\frac{D_{T}}{T_{\infty }}{\left(\frac{\partial T}{\partial r}\right)}^{2}\right)+\kappa_{hnf}\frac{1}{r}\frac{\partial }{\partial r}\left(r\;\frac{\partial T}{\partial r}\right)\\ \;+\mu_{hnf}{\left(\frac{\partial u}{\partial r}\right)}^{2}+\frac{\sigma_{f}B^{2}{}_{o}}{1+m^{2}}\;u^{2} \end{array}$$ (3)

$$u\;\frac{\partial C}{\partial x}+v\;\frac{\partial C}{\partial r}=\frac{D_{T}}{T_{\infty }}\;\frac{1}{r}\;\frac{\partial }{\partial r}\left(r\;\frac{\partial T}{\partial r}\right)+\frac{D_{B}}{r}\;\frac{\partial }{\partial r}\left(r\;\frac{\partial C}{\partial r}\right)-K^{*}\left(C-C_{\infty }\right)$$ (4)

In Eqs (1–4) the components of axial and radial velocities are respectively given by *u*, *v*. *T* is temperature, *C* is concentration, *C*_*p*_ is heat capacity, ***κ***_*hnf*_, ***μ***_*hnf*_, ***ρ***_*hnf*_ are respectively the thermal conductivity, viscosity and density of hybrid nanofluid. The temperature and concentration at free stream are *T*_∞_, *C*_∞_. The rate of dimensionless reaction is given by K* = *K*_0_/*x*. Moreover, *D*_*B*_, *D*_*T*_ are the Brownian and thermophoretic diffusion coefficients.

Subjected boundary conditions are [38]

$$\begin{array}{l} u\left(x.\;r\right)=U_{w},\;v\left(x.\;r\right)=0,\;T\left(x.\;r\right)=T_{w},\;C\left(x.\;r\right)=C_{w}\;\;\;at\;\;r=R\left(x\right),\\ u\left(x.\;r\right)\to U_{\infty },\;\;T\left(x.\;r\right)\to T_{\infty },\;C\left(x.\;r\right)\to C_{\infty }\;as\;r\to \infty \end{array}$$ (5)

To transform the modeled Eqs (1–4) we shall introduce the following group of similar variables [38]

$$\psi =\upsilon_{f}x.f\left(\eta \right),\;\eta =\frac{Ur^{2}}{\upsilon_{f}x},\;\theta \left(\eta \right)=\frac{T-T_{\infty }}{T_{w}-T_{\infty }},\;\phi \left(\eta \right)=\frac{C-C_{\infty }}{C_{w}-C_{\infty }}$$ (6)

The current study is streamlined, so in Eq (6) *ψ* is a stream function. Hence the components of flow characteristics for the assumed stream function are described as follows

$$u=\frac{1}{r}\frac{\partial \psi }{\partial r},\;v=-\frac{1}{r}\frac{\partial \psi }{\partial x}$$ (7)

In our work, the hybrid nanoprrticles are comprised of copper oxide (*CuO*) and silver (*Ag*) suspended in water (*H*_2_*O*) which is the pure fluid. For the purpose of achieving hybrid nanofluid *Ag*–*CuO*/*H*_2_*O*, first of all *CuO* -nanoparticles with volume fraction represented by *φ*_1_ are suspended in water as a result of which *CuO*/*H*_2_*O* nanofluid is obtained. Then *Ag* -nanoparticles with volume fraction *φ*_2_ are spread into *CuO*/*H*_2_*O*nanofluid as a result of this physical phenomenon *Ag* − *CuO*/*H*_2_*O* hybrid nanofluid is obtained.

After incorporating Eq (6) into Eqs (1–4) we have the following system of ODEs after simplification

$$\frac{2}{{\left(1-\varphi_{1}\right)}^{2.5}{\left(1-\varphi_{2}\right)}^{2.5}}\left(\eta f^{\prime \prime \prime }+f^{\prime \prime }\right)+\left\{\left(1-\varphi_{2}\right)\left(\left(1-\varphi_{1}\right)+\varphi_{1}\frac{\rho_{s1}}{\rho_{f}}\right)+\varphi_{2}\frac{\rho_{s2}}{\rho_{f}}\right\}ff^{\prime \prime }-\frac{M}{\left(1+m^{2}\right)}f^{\prime }=0$$ (8)

$$\begin{array}{l} 2\frac{k_{hnf}}{k_{f}}\left(\theta^{\prime }+\eta \theta^{\prime \prime }\right)+\left\{\left(1-\varphi_{2}\right)\left(\left(1-\varphi_{1}\right)+\varphi_{1}\frac{{\left(\rho C_{p}\right)}_{s1}}{{\left(\rho C_{p}\right)}_{f}}\right)+\varphi_{2}\frac{{\left(\rho C_{p}\right)}_{s2}}{{\left(\rho C_{p}\right)}_{f}}\right\}\mathrm{Pr}f\theta^{\prime }\\ +2\eta \mathrm{Pr}\left(N_{b}\theta^{\prime }\phi^{\prime }+N_{t}{\left(\theta^{\prime }\right)}^{2}\right)+\mathrm{Pr}Ec\left(\frac{\eta }{{\left(1-\varphi_{1}\right)}^{2.5}{\left(1-\varphi_{2}\right)}^{2.5}}{\left(f^{\prime \prime }\right)}^{2}+\frac{2M}{\left(1+m^{2}\right)}{\left(f^{\prime }\right)}^{2}\right)=0 \end{array}$$ (9)

$$2\left(\phi^{\prime }+\eta \phi^{\prime \prime }\right)+2\frac{N_{t}}{N_{b}}\left(\theta^{\prime }+\eta \theta^{\prime \prime }\right)+Le\phi^{\prime }-\frac{1}{2}LeK\phi =0$$ (10)

The dimensionless form of subjected BCs is

$$\begin{array}{l} f\left(c\right)=\frac{\varepsilon }{2}c,\;f^{\prime }\left(c\right)=\frac{\varepsilon }{2},\;\theta \left(c\right)=1,\;\phi \left(c\right)=1\\ f^{\prime }\left(\infty \right)\to \frac{1}{2}\left(1-\varepsilon \right),\;\theta \left(\infty \right)\to 0,\;\phi \left(\infty \right)\to 0 \end{array}$$ (11)

It is to be noticed that above *κ*_*s*1_, *ρ*_*s*1_, (*ρC*_*p*_)_*s*1_, *φ*_1_ are respectively the thermal conductivity, density, heat capacity and volume fraction for *CuO* -nanoparticles, while *κ*_*s*2_, *ρ*_*s*2_, (*ρC*_*p*_)_*s*2_, *φ*_2_ are similar notations for *Ag* -nanoparticles. Moreover, in Eqs (8–11) we have some substantial parameters which are given in Table 1 along with mathematical description and physical interpretation.

**Table 1:** Information of emerging parameters.

Symbolic notation	Mathematical notation	Physical meaning
Pr	*V*_*f*_(*ρC*_*p*_)_*hnf*_/*k*_*f*_	Prandtl number
*N*_*b*_	***τ****D*_*B*_(*C*_*w*_-*C*_∞_)/*v*_*f*_	Brownian motion parameter
*N*_*t*_	***τ****D*_*T*_(*T*_*w*_-*T*_∞_)/*T*_∞_*v*_*f*_	Thermophoretic parameter
*Ec*	*U*^2^/(*T*_*w*_-*T*_∞_)*C*_*p*_	Eckert number
*M*	*σB*_0_^2^*x*/2*Uv*_*f*_	Magnetic parameter
*Le*	*v*_*f*_/*D*_*B*_	Lewis number
*K*	K*/*U*	Chemical reaction parameter
*ε*	*u*_*w*_/*U*	Velocity ratio parameter

It is to be noticed that the chemical reaction parameter *K* < 0 depicts a generation chemical reaction while *K* > 0 describes a destructive chemical reaction. Moreover, the parameter *ε* = *u*_*w*_/*U* represents an important characteristic for flow system which is described as (i) when *ε* = 1 then fluid is static but needle is moving (ii) when *ε* = 0 then fluid is moving but needle is static (iii) when 0 < *ε* < 1 then needle and fluid are moving in similar direction. The thermophysis characteristics for nanofluid and hybrid nanofluid are described in Table 2, while their numerical expressions are given in Table 3.

**Table 2:** Themophoresis properties of hybrid nanofluid [3, 39].

Properties	Nanofluid (*CuO*)
**Density**	*ρ*_*nf*_ = (1-*ϕ*_1_)*ρ*_*f*_ + *ϕ*_1_*ρ*_*s*1_
**Heat Capacity**	(*ρC*_*p*_)_*nf*_ = (1-*ϕ*_1_)(*ρC*_*p*_)_*f*_ + *ϕ*_1_(*ρC*_*p*_)_*s*1_
**Viscosity**	$\mu_{nf}=\frac{\mu_{f}}{{\left(1-\varphi_{1}\right)}^{2.5}}$
**Thermal Conductivity**	$\kappa_{nf}=\frac{\kappa_{s1}+2\;\kappa_{f}-2\varphi_{1}\;\left(\kappa_{f}-\kappa_{s1}\right)}{\kappa_{s1}+2\;\kappa_{f}+\varphi_{1}\;\left(\kappa_{f}-\kappa_{s1}\right)}\kappa_{f}$
**Hybrid Nanofluid** (*Ag* − *CuO*)	
**Density**	$\rho_{hnf}=\left\{\left(1-\varphi_{2}\right)\left(\left(1-\varphi_{1}\right)+\varphi_{1}\frac{\rho_{s1}}{\rho_{f}}\right)+\varphi_{2}\frac{\rho_{s2}}{\rho_{f}}\right\}\rho_{f}$
**Heat Capacity**	${\left(\rho C_{p}\right)}_{hnf}=\left\{\left(1-\varphi_{2}\right)\left(\left(1-\varphi_{1}\right)+\varphi_{1}\frac{{\left(\rho C_{p}\right)}_{s1}}{{\left(\rho C_{p}\right)}_{f}}\right)+\varphi_{2}\frac{{\left(\rho C_{p}\right)}_{s2}}{{\left(\rho C_{p}\right)}_{f}}\right\}{\left(\rho C_{p}\right)}_{f}$
**Viscosity**	$\mu_{hnf}=\frac{\mu_{f}}{{\left(1-\varphi_{1}\right)}^{2.5}{\left(1-\varphi_{2}\right)}^{2.5}}$
**Thermal Conductivity**	$\kappa_{hnf}=\frac{\kappa_{s2}+2\kappa_{f}-2\varphi_{2}\left(\kappa_{f}-\kappa_{s2}\right)}{\kappa_{s2}+2\kappa_{f}+\varphi_{2}\left(\kappa_{f}-\kappa_{s2}\right)}\kappa_{nf}$

**Table 3:** Numerical values of nanoparticles and base fluid for thermophysical properties [39].

Thermophysical Properties	(*Silver* -*Ag*)	(*Copper Oxide* -*CuO*)	(*Water*–*H*_2_*O*)
	Nanoparticles	Nanoparticles	Base Fluid
*ρ*(*kg*/*m*^3^)	10500	6320	997.1
*C*_*p*_(*J*/*kg* K)	235	531.8	4179
*κ* (*W*/ *mK*)	429	76.5	0.613

### 2.3 Main quantities of interest

The coefficient of skin friction, local Nusselt and Sherwood numbers for our flow system are described as

$$C_{f}=\frac{\mu_{hnf}}{\rho_{f}u_{w}{}^{2}}{\left(\frac{\partial u}{\partial r}\right)}_{r=R},\;Nu_{x}=-\frac{x\kappa_{hnf}}{k_{f}\left(T_{w}-T_{\infty }\right)}{\left(\frac{\partial T}{\partial r}\right)}_{r=R},\;Sh_{x}=\frac{-x}{\left(C_{w}-C_{\infty }\right)}{\left(\frac{\partial C}{\partial r}\right)}_{r=R}$$ (12)

After incorporating Eq (6)) in Eq (12) we have these physical quantities in dimensionless form as given below

$$\begin{array}{l} C_{f}{\text{Re}}_{x}{}^{1/2}=4c^{1/2}\frac{1}{{\left(1-\varphi_{1}\right)}^{2.5}{\left(1-\varphi_{2}\right)}^{2.5}}f^{\prime \prime }\left(c\right),\\ \;\;Nu_{x}{\text{Re}}_{x}{}^{-1/2}=-2\frac{\kappa_{hnf}}{\kappa_{f}}c^{1/2}\theta^{\prime }\left(c\right),\;\\ \;\;\;Sh_{x}{\text{Re}}_{x}{}^{1/2}=-2c^{1/2}\phi^{\prime }\left(c\right) \end{array}$$ (13)

In Eq (13) ${\text{Re}}_{x}=\frac{Ux}{\upsilon_{f}}$ depicts local Reynolds number.

## 3. Solution of problem

In order to determine the solution for dimensionless set of Eqs (8–10) by incorporating the boundary conditions as given in Eq (11) we shall use the semi analytical technique HAM [40, 41]. To employ this method we need some initial guess for solution of Eqs (8–10), these initial guesses are stated below

$$f_{0}\left(\eta \right)=1-e^{\eta },\;\Theta_{0}\left(\eta \right)=\frac{\gamma_{1}}{1+\gamma_{1}}e^{-\eta },\;\Phi_{0}\left(\eta \right)=\frac{\gamma_{2}}{1+\gamma_{2}}e^{-\eta }$$ (14)

The linear operators are defined as follows

$$L_{f}\left(f\right)=f^{\prime \prime \prime }-f^{\prime },\;L_{\Theta }\left(\Theta \right)=\theta^{\prime \prime }-\theta ,\;L_{\Phi }\left(\Phi \right)=\phi^{\prime \prime }-\phi$$ (15)

It is to be noticed that the expanded form of operators stated in Eq (15) is defined as follows

$$L_{f}\left(a_{1}+a_{2}e^{\eta }+a_{3}e^{-\eta }\right)=0,\;L_{\Theta }\left(a_{4}e^{\eta }+a_{5}e^{-\eta }\right)=0,\;L_{\Phi }\left(a_{6}e^{\eta }+a_{7}e^{-\eta }\right)=0$$ (16)

In Eq (16) *a*_*i*_ *for i* = 1,2,3,…‥7 are considered as constants.

By employing the Taylor series expansion we have

$$\begin{array}{l} f(\eta ;\;\zeta )=f_{0}(\eta )+\sum_{n=1}^{\infty }f_{n}\;(\eta )\zeta^{n}\\ \Theta \;(\eta ;\;\zeta )=\Theta_{0}\;(\eta )+\sum_{n=1}^{\infty }\Theta_{n}\;(\eta \;)\;\zeta^{n}\\ \Phi \;(\eta ;\;\zeta )=\Phi_{0}\;(\eta )+\sum_{n=1}^{\infty }\Phi_{n}\;(\eta )\;\zeta^{n} \end{array}$$ (17)

## 4. Results and discussion

This work, addresses the capability to administer the distribution of heat transmission for fluid flow occurs upon a paraboloid thin shaped hot needle by using hybrid nanoparicles containing Silver and Copper Oxide with water as base/pure fluid. The needle is placed horizontally in nanofluid using of Hall current and viscous dissipation. HAM is used to determine the solution for modeled problem. The impact of various emerging parameters will be discussed next.

### 4.1 Flow characteristics

**Fig 2:**
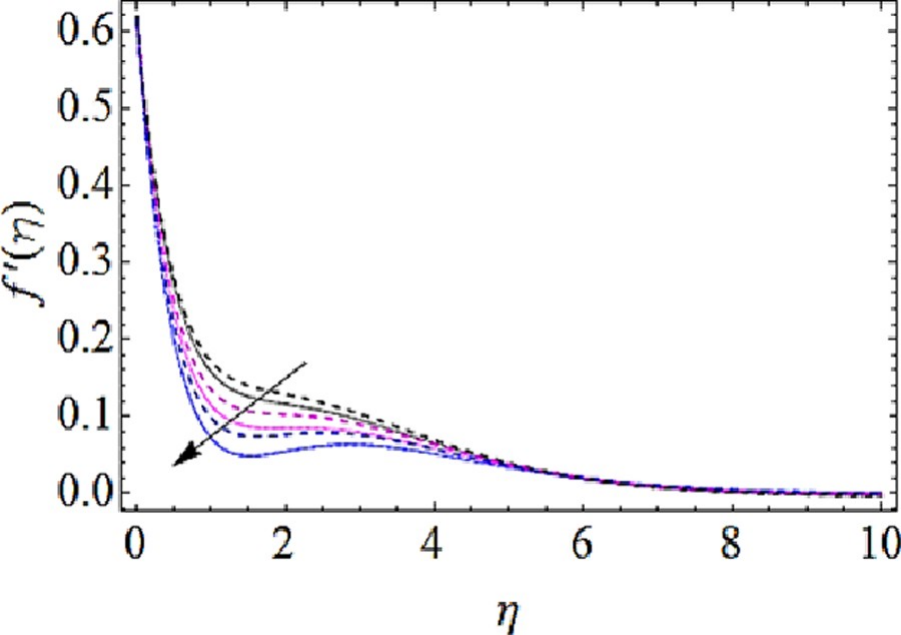
Velocity profiles for different values of magnetic parameter.

**Fig 3:**
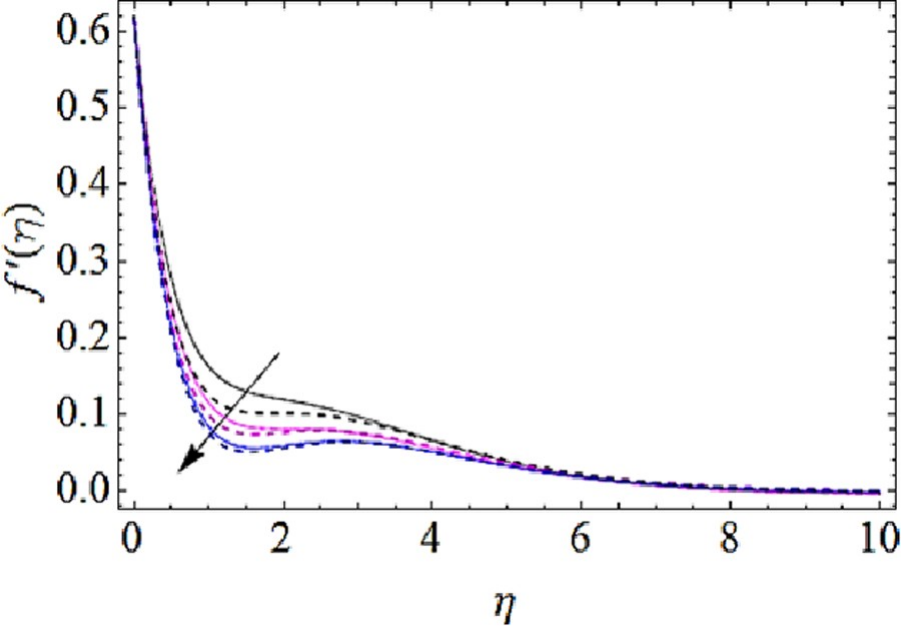
Velocity profiles for different values of CuO volume fraction.

**Fig 4:**
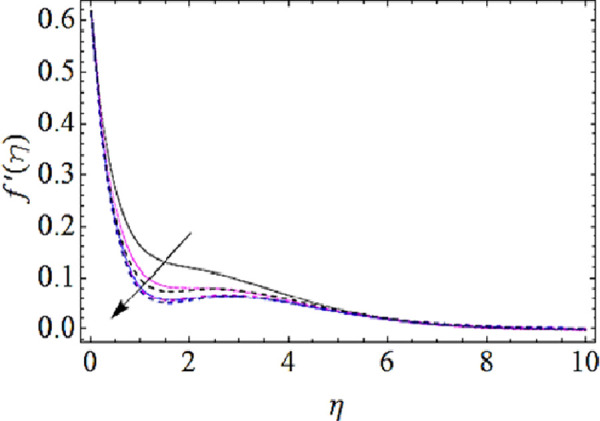
Velocity profiles for different values of Ag volume fraction.

In this subsection we are to talk about the effects of magnetic parameter (*M*), volume fractions *φ*_1_(for *CuO*-nanoparticles) and *φ*_1_(for *Ag*-nanoparticles) upon flow characteristics as shown in Figs 2–4. From Fig 2 we observed that flow of fluid reduces with augmenting values of magnetic field. Actually application of magnetic effects to flow system results in generation of Lorentz force that opposes the velocity of flow system and hence velocity reduces with augmentation in magnetic parameter (*M*). From this physical phenomenon it is also observed that magnetic field plays a vital role in controlling flow characteristics of nanofluid. Figs 3 and 4 depict the impact of volume fractions *φ*_1_ and *φ*_1_ upon flow of nanofluid. It is to be noticed that with raise in volume fractions of nanoparticles the viscosity of the liquid also enhances. Due to the augmentation of viscous forces the flow of fluid reduces as shown in Figs 3 and 4.

### 4.2 Thermal characteristics

**Fig 5:**
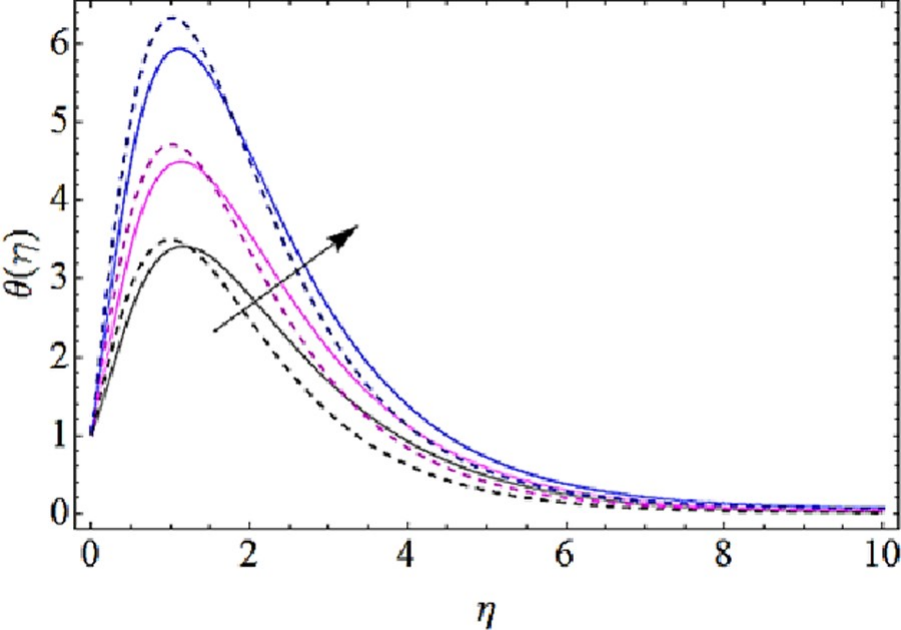
Thermal characteristics for different values of Eckert number.

**Fig 6:**
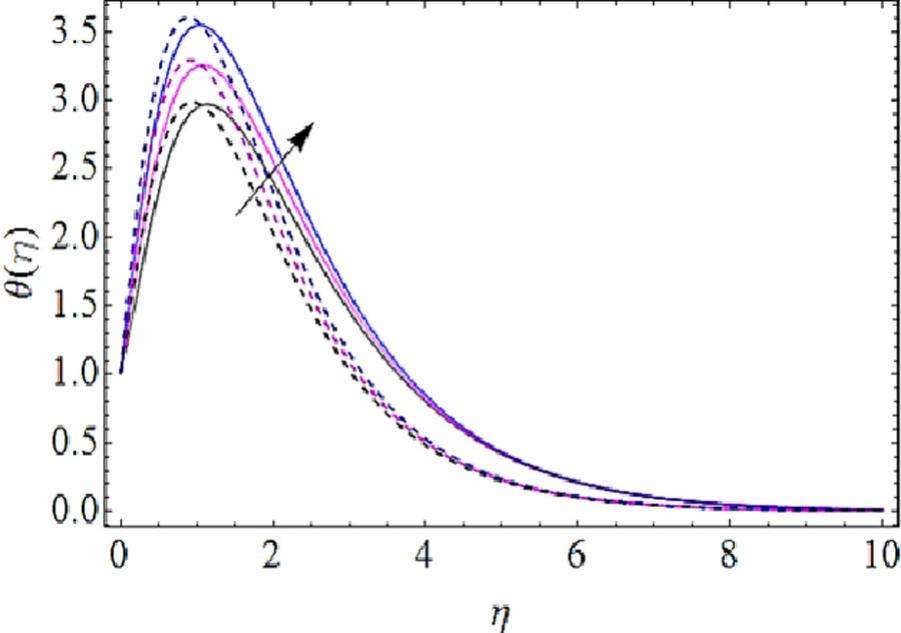
Thermal characteristics for different values of magnetic parameter.

**Fig 7:**
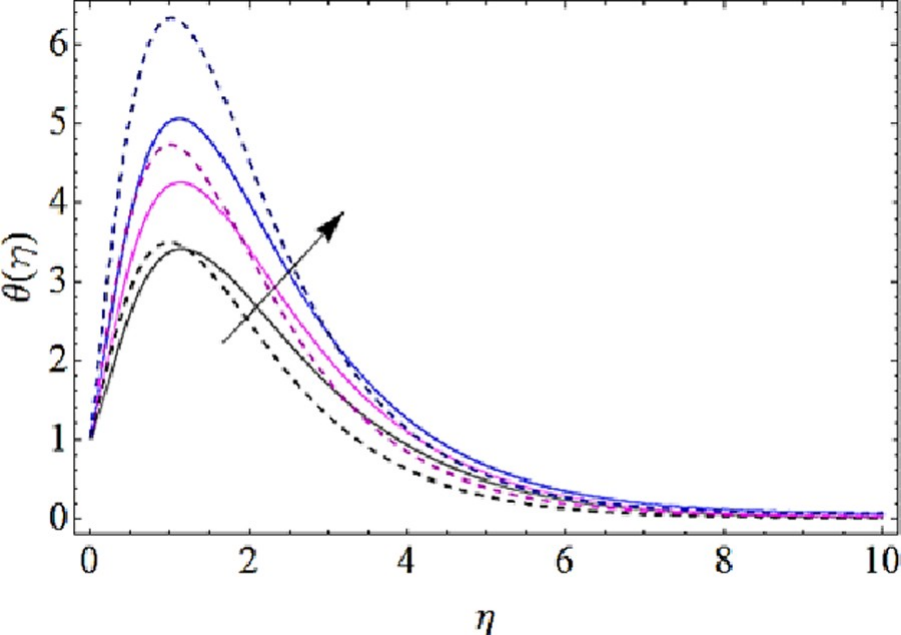
Thermal characteristics for different values of CuO volume fraction.

**Fig 8:**
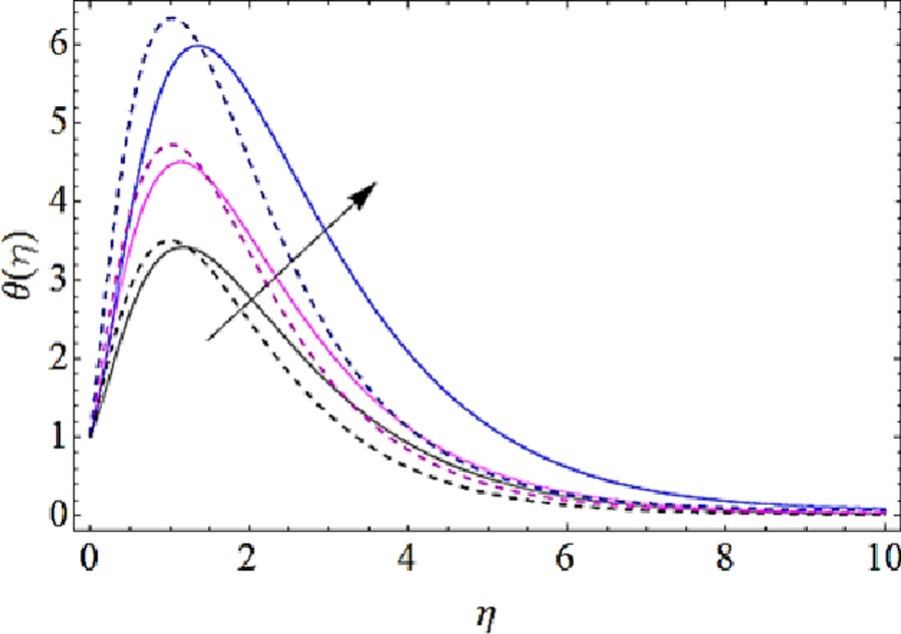
Thermal characteristics for different values of Ag volume fraction.

**Fig 9:**
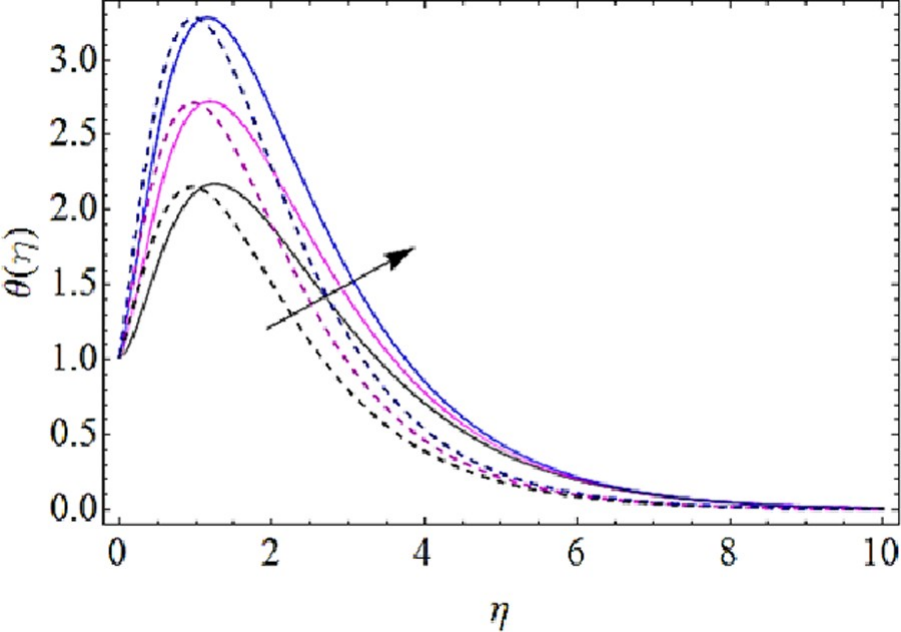
Thermal characteristics for different values of Brownian motion parameter.

**Fig 10:**
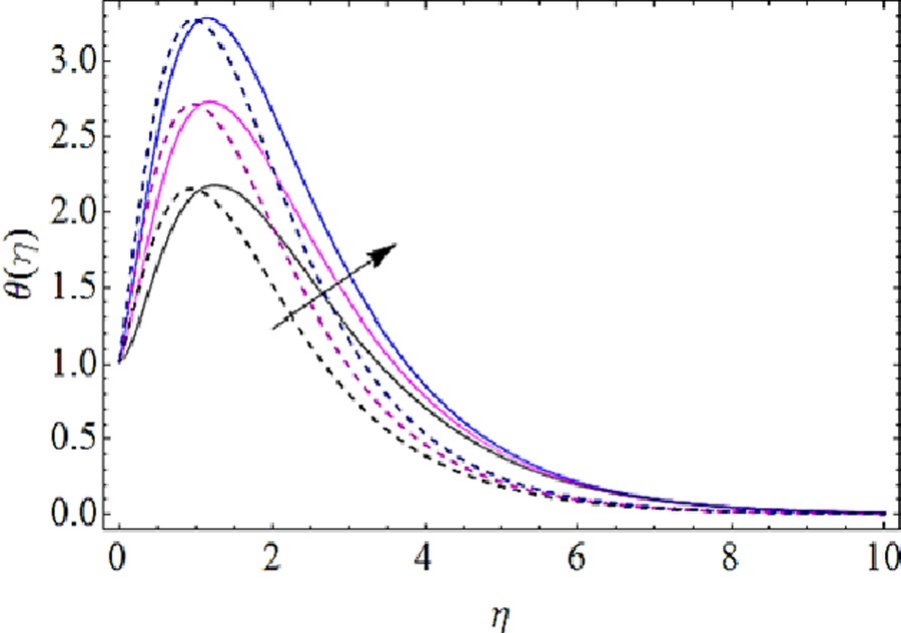
Thermal characteristics for different values of thermophoresis parameter.

**Fig 11:**
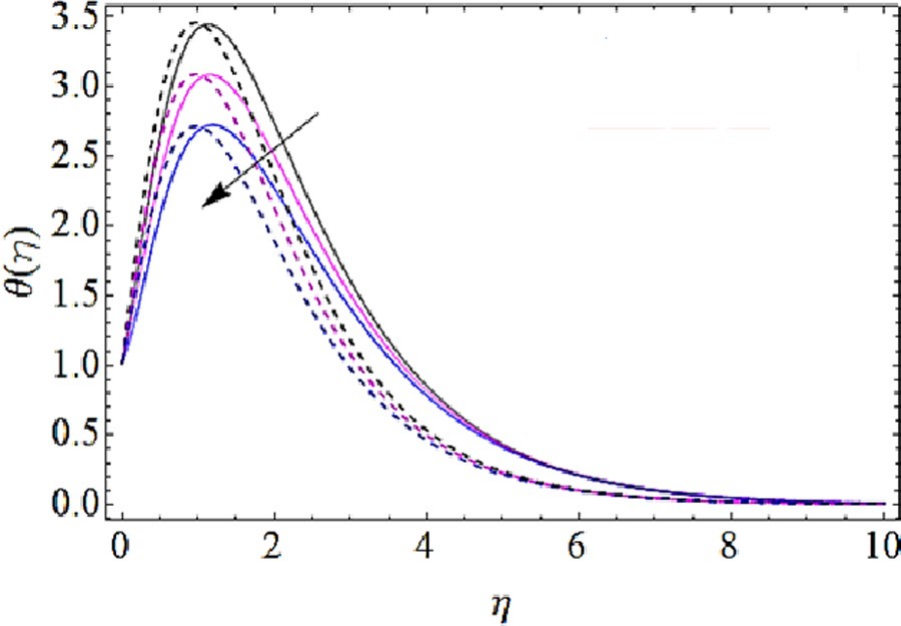
Thermal characteristics for different values of Prandtl number.

Next we shall highlight the influence of Eckert number (*Ec*), Magnetic parameter (*M*), volume fractions *φ*_1_(for *CuO*-nanoparticles),*φ*_2_(for *Ag*-nanoparticles), Brownian motion parameter (*N*_*b*_), thermophoresis parameter (*N*_*t*_) and Prandtl number (Pr) upon temperature of fluid as shown in Figs 5–11. From Fig 5 it is observed that due to the augmentation in (*Ec*) there is an enhancement in the transportation of thermal energy and finally enhances the hotness of nanofluid. From Fig 6 we see that the growth in (*M*) enhances the conduction of energy in nanoparticles of hybrid nanofluid. Due to this physical phenomenon Lorentz force augments and finally increases the thermal boundary layer. Hence increase in magnetic parameter enhances the thermal characteristics of nanofluid. Since with increase in volume fractions of silver and copper oxide nanoparticles, there is a corresponding increase in dense behavior of nanofluid that will enhance the thermal boundary layer thickness of fluid. Hence augmentation in volume fractions enhances thermal characteristics of the nanofluid as directed in Figs 7 and 8. With growth in (*N*_*b*_), there is a boost in the random motion of nanoparticles which leads to the enhancement of collision amongst these nanoparticles. Due to this physical phenomenon, the kinetic energy of the nanoparticles is transmitted to heat energy and grows up the thermal boundary layer of fluid as depicted in Fig 9. The impact of thermophoresis parameter (*N*_*t*_) upon thermal characteristics is shown in Fig 10. Since *N*_*t*_ = ***τ****D*_*T*_ (*T*_*w*_—*T*_∞_)/*T*_∞_*v*_*f*_, so for increase in (*N*_*t*_), there will be an augmentation in temperature gradient of nanofluid. Hence for larger values of (*N*_*t*_), maximum heat will transfer as presented in Fig 10. Moreover, with augmentation in Prandtl number there is a reduction in mass as well as thermal diffusivity of the nanoparticles. So with increase in Prandtl number we have a reduction in thermal characteristics of the fluid as shown in Fig 11

### 4.3 Concentration characteristics

**Fig 12:**
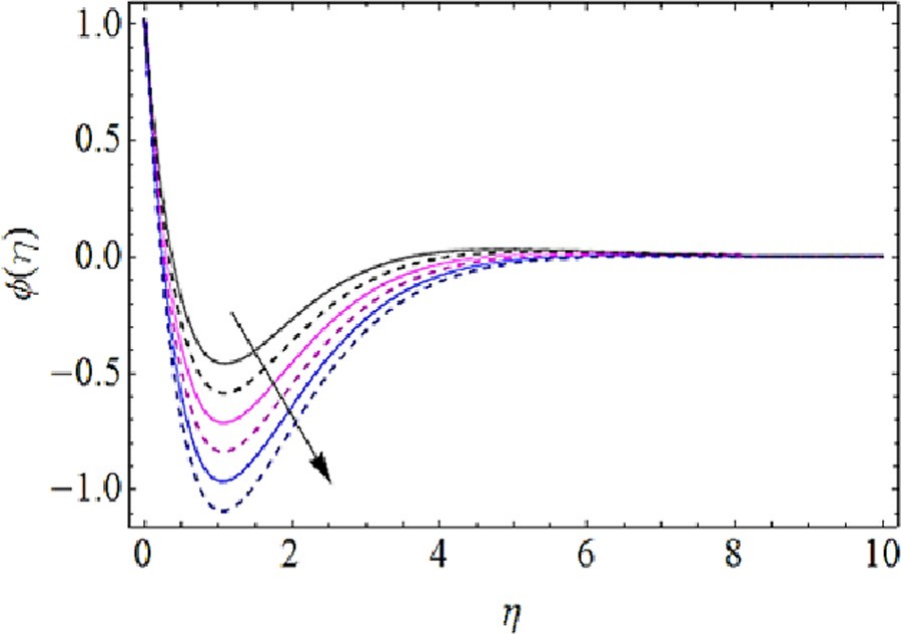
Concentration characteristics for various values of Lewis number.

**Fig 13:**
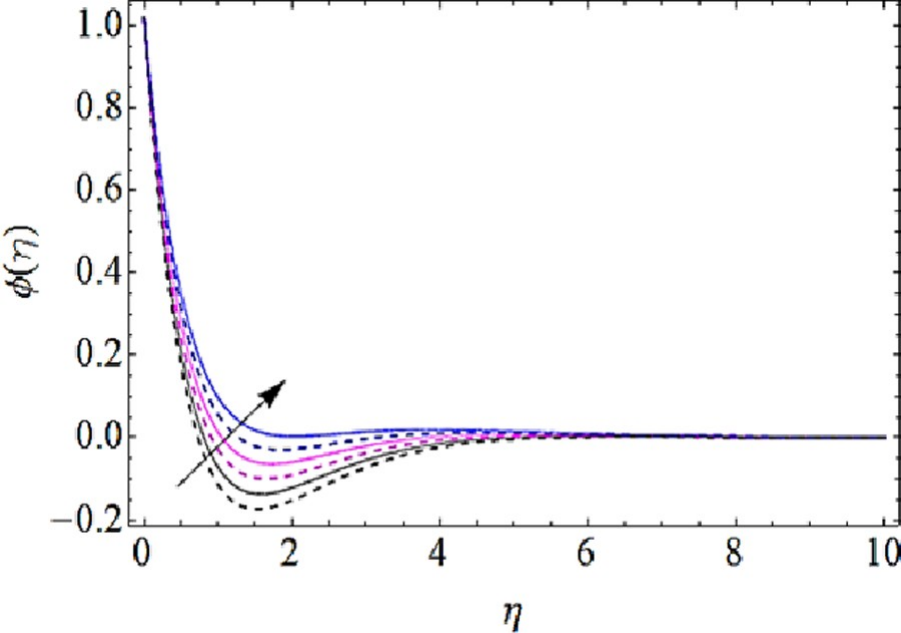
Concentration characteristics for various values of Brownian motion parameter.

**Fig 14:**
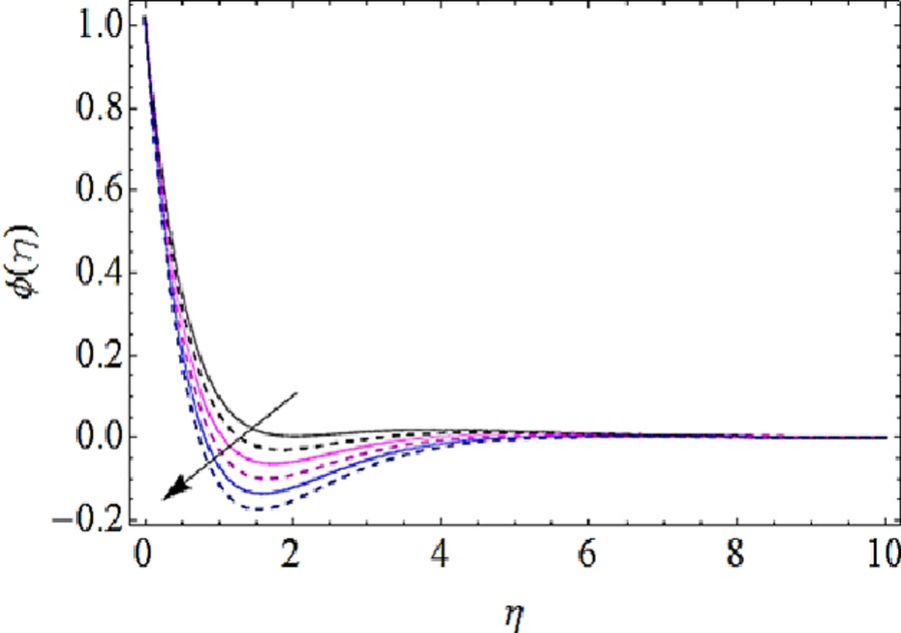
Concentration characteristics for various values of thermophoresis parameter.

**Fig 15:**
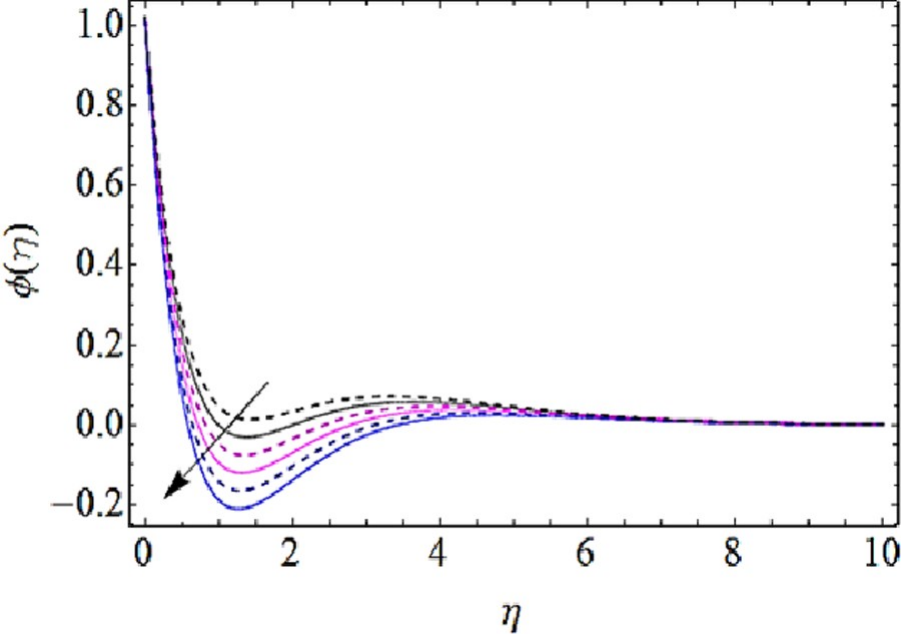
Concentration characteristics for various values of chemical reaction parameter.

Next we shall discuss the impact of *Le*, *N*_*b*_, *N*_*t*_, *K* upon concentration characteristics with the help of graphs as shown in Figs 12–15. Since with increase in Lewis number, there is a reduction in mass as well thermal diffusivities of nanofluid. Hence growth in Lewis number weakens the concentration boundary layer and hence reduces the concentration profile as shown in Fig 12. The increase in *N*_*b*_ reduces the rate of mass transmission, hence boundary layer thickness of nanofluid enhances as shown in Fig 13. On the other hand the augmentation in thermophoresis parameter results in increase of thermal conductivity of the nanoparticles that infiltrates deeper inside the nanofluid and ultimately reduces the thickness of concentration boundary layer as presented in Fig 14. Finally, with augmentation in chemical reaction parameter the thickness of concentration boundary layer reduces. Actually the presence of chemical reaction declines the concentration boundary layer; hence growing values *K* reduces the concentration characteristics of nanofluid as depicted in Fig 15.

### 4.4 Table discussion

Next we shall discuss the impact of various substantial parameters numerically upon skin friction coefficient, Nusselt and Sherwood numbers as presented in Tables 4–6. From these tables we observe that the size of needle greatly affected the above stated physical quantities. It is also revealed from these tables that the skin friction reduces whereas Nusselt and Sherwood numbers expand for increasing values of emerging parameter (*ε*). Table 4 depicts numerically the impact of Needle’s size, velocity ratio and magnetic parameters upon skin friction coefficient. Due to increase in resistive forces for corresponding increase in magnetic parameter the values of friction also enhances as shown in Table 3. Similarly with increase in Prandtl number the thermal characteristics reduce and hence Nusselt number drops down, on the other hand with growth in Eckert number the thermal dissipation enhances that result in augmentation of Nusselt number as presented in Table 5. In Table 6 influence of various emerging parameters is presented numerically.

**Table 4:** Impact upon skin friction coefficient *f”*(*c*) when Pr = 7, *ϕ*_1_ = 0.02 = *ϕ*_2_, *m* = 0.4.

*c*	*ε*	*M*	*f”*(*c*) *For CuO*	*f”*(*c*) *For CuO + Ag*
0.002	0.1	0.2	0.4132580	0.4262724
0.02			0.4241918	0.4324283
0.1			0.4744767	0.4847955
	0.2		0.2991480	0.3193390
	0.3		0.1168840	0.2141966
		0.3	0.3487980	0.3545914
		0.4	0.3916890	0.4152412

**Table 5:** Impact of different parameters on heat transfer coefficient *θ’*(*c*).

*c*	*ε*	*ϕ*_1_ = *ϕ*_2_	*M*	*Ec*	*Nb*	*Nt*	*θ’*(*c*) *For CuO*	*θ’*(*c*) *For CuO + Ag*
0.002	0.1	0.01	0.2	2	0.1	0.1	7.78790	8.83699
0.02							7.83978	8.99425
0.1							8.07489	9.84849
	0.2						8.79554	8.92995
	0.3						8.89984	8.95792
		0.02					9.11929	9.33258
		0.03					9.63384	9.81129
			0.3				9.64384	9.70977
			0.4				9.764432	9.81992
				5			10.23417	10.57983
				7			10.44528	10.69945
					0.2		9.653321	9.60981
					0.3		9.54321	9.49870
						0.2	9.43209	9.38760
						0.3	9.32108	9.27650

**Table 6:** Influence of various parameters on coefficient of the Sherwood *ϕ’*(*c*) when Pr = 7.

*c*	*ε*	*Nt*	*Nb*	*ϕ’*(*c*) *For CuO*	*ϕ’*(*c*) *For CuO + Ag*
0.002	0.1	0.1	0.2	2.36446	2.384200
0.02				2.37760	2.477630
0.2				2.51585	2.515970
	0.2			2.71848	2.719510
	0.3			2.52108	2.543410
		0.2		2.42106	2.352122
		0.3		2.31015	2.252610
			0.3	2.543410	2.654521
			0.4	2.687621	2.876510

## 5. Conclusions

In this work the heat transmission for fluid flow occurs upon a paraboloid thin shaped hot needle by using hybrid nanoparicles containing Silver and Copper Oxide with water as base/pure fluid. The needle is placed horizontally in nanofluid in the presence of some physical conditions. HAM is used for determination of solution for modeled problem. The analytical investigation has carried out for current problem. The impact of various emerging parameters upon flow, thermal and concentration characteristics has discussed with the help of graphical views. After detail study of the work the following points are highlighted:-

- The application of magnetic effects to flow system results in generation of Lorentz force that opposes the velocity of flow system and hence velocity reduces in this physical phenomenon.
- With augmentation in volume fractions of nanoparticles the viscosity of the nanofluid also enhances that declines the flow of fluid.
- With growth in Eckert number and magnetic parameter there is an enhancement in temperature profile.
- The augmenting values of magnetic parameter results in Lorentz force that leads to an augmentation of thermal boundary layer thickness which augments the temperature of flow system.
- Increase in volume fractions of silver and copper oxide nanoparticles enhances the viscous behavior of nanofluid and results in enhancement of the thermal characteristics of the nanofluid.
- With increase in Brownian motion and thermophoresis parameters there is a growth in temperature profile. On the other hand growth in Prandtl number reduces the thickness of thermal boundary layer.
- The growth in Lewis number reduces mass and heat diffusivities of nanofluid and ultimately weakens the concentration boundary layer thickness that results in reduction of concentration profile.
- The enhancement in Brownian motion reduces the mass transmission rate as a result of which the thickness of concertation boundary layer of nanofluid enhances and hence the concentration profile of nanoparticles increases.
- The growing values of thermophoresis parameter increase the thermal conductivity of nanoparticles that infiltrates deeper in nanofluid and hence concentration profile of nanoparticles reduces.
- Growth in chemical reaction parameter reduces the concentration characteristics of nanofluid.

## Supporting information

S1 Nomenclature (DOCX)

## References

[1] Choi SUS (1995) Enhancing thermal conductivity of fluids with nanoparticles. ASME Int Mech Eng 66: 99–105.

[2] Hayat T, Khan MI, Farooq M, Yasmeen T, Alsaedi A (2016) Water-carbon nanofluid flow with variable heat flux by a thin needle. Journal of Molecular Liquids, 224, 786–791.

[3] Waini I, Ishak A, Pop I (2019) Hybrid nanofluid flow and heat transfer past a vertical thin needle with prescribed surface heat flux. International Journal of Numerical Methods for Heat & Fluid Flow 29 (12), 4875–4894.

[4] Khan WA, Ali M, Irfan M, Khan M, Shahzad M, Sultan F (2019) A rheological analysis of nanofluid subjected to melting heat transport characteristics. Applied Nanoscience, 1–10.

[5] Waini I, Ishak A, Pop I (2020) Hybrid nanofluid flow past a permeable moving thin needle. Mathematics, 8(4), 612.

[6] Krishna PM, Sharma RP, Sandeep N (2017) Boundary layer analysis of persistent moving horizontal needle in Blasius and Sakiadis magnetohydrodynamic radiative nanofluid flows. Nuclear Engineering and Technology, 49(8), 1654–1659.

[7] Khan M, Khan WA, Alshomrani AS (2016) Non-linear radiative flow of three-dimensional Burgers nanofluid with new mass flux effect. International Journal of Heat and Mass Transfer, 101, 570–576.

[8] Khan WA, Irfan M, Khan M, Alshomrani AS, Alzahrani AK, Alghamdi MS (2017) Impact of chemical processes on magneto nanoparticle for the generalized Burgers fluid. Journal of Molecular Liquids, 234, 201–208.

[9] Khan WA, Khan M, Malik R (2014) Three-dimensional flow of an Oldroyd-B nanofluid towards stretching surface with heat generation/absorption. PLoS One, 9(8), e105107. 10.1371/journal.pone.0105107 25170945 PMC4149422

[10] Khan WA, Sultan F, Ali M, Shahzad M, Khan M, Irfan M (2019) Consequences of activation energy and binary chemical reaction for 3D flow of Cross-nanofluid with radiative heat transfer. Journal of the Brazilian Society of Mechanical Sciences and Engineering, 41(1), 1–13.

[11] Al-Hossainy AF, Eid MR (2020) Structure, DFT calculations and heat transfer enhancement in [ZnO/PG+ H 2 O] C hybrid nanofluid flow as a potential solar cell coolant application in a double-tube. Journal of Materials Science: Materials in Electronics, 31(18), 15243–15257.

[12] Eid MR, Al-Hossainy AF (2021) Combined experimental thin film, DFT-TDDFT computational study, flow and heat transfer in [PG-MoS2/ZrO2] C hybrid nanofluid. Waves in Random and Complex Media, 1–26.

[13] Al-Hossainy AF, Eid MR (2021) Combined Experimental Thin Films, TDDFT-DFT Theoretical Method, and Spin Effect on [PEG-H2O/ZrO2+ MgO] h Hybrid Nanofluid Flow with Higher Chemical Rate. Surfaces and Interfaces, 100971.

[14] Eid MR, Mabood F, Mahny KL (2020) On 3D Prandtl nanofluid flow with higher-order chemical reaction. Proceedings of the Institution of Mechanical Engineers, Part C: Journal of Mechanical Engineering Science, 0954406220975429.

[15] Alaidrous AA, Eid MR (2020) 3-D electromagnetic radiative non-Newtonian nanofluid flow with Joule heating and higher-order reactions in porous materials. Scientific Reports, 10(1), 1–19. 10.1038/s41598-019-56847-4 32884033 PMC7471956

[16] Eid MR, Nafe MA (2020) Thermal conductivity variation and heat generation effects on magneto-hybrid nanofluid flow in a porous medium with slip condition. Waves in Random and Complex Media, 1–25.

[17] Lee LL (1967) Boundary layer over a thin needle, Phys. Fluids 10, 820–822.

[18] Narain JP, Uberoi MS (1972) Forced heat transfer over a thin needle, J. Heat Transfer 94, 240–242.

[19] Narain JP, Uberoi MS (1972) Free-convection heat transfer from a thin vertical needle, Phys. Fluids 15, 928–929.

[20] Upreti H, Kumar M (2019) Influence of non-linear radiation, Joule heating and viscous dissipation on the boundary layer flow of MHD nanofluid flow over a thin moving needle. Multidiscipline Modeling in Materials and Structures.

[21] Sulochana C, Ashwinkumar GP, Sandeep N (2018) Boundary layer analysis of persistent moving horizontal needle in magnetohydrodynamic ferrofluid: A numerical study. Alexandria engineering journal, 57(4), 2559–2566.

[22] Khan WA, Ali M, Sultan F, Shahzad M, Khan M, Irfan M (2019) Numerical interpretation of autocatalysis chemical reaction for nonlinear radiative 3D flow of cross magnetofluid. Pramana, 92(2), 16.

[23] Khan WA, Ali M (2019) Recent developments in modeling and simulation of entropy generation for dissipative cross material with quartic autocatalysis. Applied Physics A, 125(6), 1–9.

[24] Khan WA, Ali M, Waqas M, Shahzad M, Sultan F, Irfan M (2019) Importance of convective heat transfer in flow of non-Newtonian nanofluid featuring Brownian and thermophoretic diffusions. International Journal of Numerical Methods for Heat & Fluid Flow.

[25] Mabood F, Shateyi S, Rashidi MM, Momoniat E, Freidoonimehr N (2016) MHD stagnation point flow heat and mass transfer of nanofluids in porous medium with radiation, viscous dissipation and chemical reaction. Adv. Powder Technol. 27, 742–749.

[26] Ramzan M, Shaheen N, Kadry S, Ratha Y, Nam Y (2020) Thermally Stratified Darcy Forchheimer Flow on a Moving Thin Needle with Homogeneous Heterogeneous Reactions and Non-Uniform Heat Source/Sink. Applied Sciences, 10(2), 432.

[27] Khan MI, Waqas M, Hayat T, Alsaedi A (2017) A comparative study of Casson fluid with homogeneous-heterogeneous reactions. Journal of colloid and interface science, 498, 85–90. 10.1016/j.jcis.2017.03.024 28319844

[28] Makinde OD, Animasaun IL (2016) Thermophoresis and Brownian motion effects on MHD bioconvection of nanofluid with nonlinear thermal radiation and quartic chemical reaction past an upper horizontal surface of a paraboloid of revolution. Journal of Molecular liquids, 221, 733–743.

[29] Hamid A (2020) Terrific effects of Ohmic-viscous dissipation on Casson nanofluid flow over a vertical thin needle: buoyancy assisting & opposing flow. Journal of Materials Research and Technology, 9(5), 11220–11230.

[30] Eid MR, Mahny KL, Dar A, Muhammad T (2020) Numerical study for Carreau nanofluid flow over a convectively heated nonlinear stretching surface with chemically reactive species. Physica A: Statistical Mechanics and its Applications, 540, 123063.

[31] Ghiasi EK, Saleh R (2019) Analytical and numerical solutions to the 2D Sakiadis flow of Casson fluid with cross diffusion, inclined magnetic force, viscous dissipation and thermal radiation based on Buongiorno’s mathematical model. CFD Lett, 11(1), 40–54.

[32] Raju SS, Kumar KG, Rahimi-Gorji M, Khan I (2019) Darcy–Forchheimer flow and heat transfer augmentation of a viscoelastic fluid over an incessant moving needle in the presence of viscous dissipation. Microsystem Technologies, 25(9), 3399–3405.

[33] Mishra A, Kumar M (2019) Viscous dissipation and Joule heating influences past a stretching sheet in a porous medium with thermal radiation saturated by silver–water and copper–water nanofluids. Special Topics & Reviews in Porous Media: An International Journal, 10(2), 171–186.

[34] Farooq U, Afridi MI, Qasim M, Lu DL (2018) Transpiration and viscous dissipation effects on entropy generation in hybrid nanofluid flow over a nonlinear radially stretching disk. Entropy, 20(9), 668. 10.3390/e20090668 PMC7513191 33265757

[35] Alotaibi H, Althubiti S, Eid MR, Mahny KL (2020) Numerical treatment of mhd flow of casson nanofluid via convectively heated non-linear extending surface with viscous dissipation and suction/injection effects. Computers, Materials & Continua, 66(1), 229–245.

[36] Ahmad R, Mustafa M, Hina S (2017) Buongiorno’s model for fluid flow around a moving thin needle in a flowing nanofluid: A numerical study. Chinese journal of physics, 55(4), 1264–1274.

[37] Soid SK, Ishak A, Pop I (2017) Boundary layer flow past a continuously moving thin needle in a nanofluid. Applied Thermal Engineering, 114, 58–64.

[38] Mabood F, Nayak MK, Chamkha AJ (2019) Heat transfer on the cross flow of micropolar fluids over a thin needle moving in a parallel stream influenced by binary chemical reaction and Arrhenius activation energy. The European Physical Journal Plus, 134(9), 427.

[39] Hayat T, Nadeem S (2017) Heat transfer enhancement with Ag–CuO/water hybrid nanofluid. Results in physics, 7, 2317–2324.

[40] Liao SJ (1999) Explicit totally analytic approximate solution for blasius viscous flow problems, Int. J. Non-Linear Mech. 34, 759–778.

[41] Liao SJ (2003) On the analytic solution of Magnetohydrodynamic Flows of Non-Newtonian fluids over a stretching sheet, J. Fluid Mech. 488, 189–212.

