# Supplementary material for: Chemically reactive nanofluid flow past a thin moving needle with viscous dissipation, magnetic effects and hall current
Source: PLoS One. 2021 Apr 15;16(4):e0249264. doi: 10.1371/journal.pone.0249264 (PMC8049295; doi:10.1371/journal.pone.0249264)
Supplement: S1 Nomenclature — (DOCX) [file pone.0249264.s001.docx]

**S1 Nomenclature.**

| Symbol |  | Description |  | Symbol |  | Description |
| --- | --- | --- | --- | --- | --- | --- |
| $T_{w}$ |  | Wall temperature$\left(K\right)$ |  | $Le$ |  | Lewis number |
| $T_{\infty}$ |  | Fluid temperature at free stream$\left(K\right)$ |  | $Ec$ |  | Eckert number |
| $M$ |  | Magnetic parameter |  | $K$ |  | Chemical reaction parameter |
| $u_{w}$ |  | Needle velocity |  | $u_{\infty}$ |  | Immersed fluid velocity |
| $C_{w}$ |  | Wall concentration |  | $P_{e}$ |  | Peclet number |
| $C_{\infty}$ |  | Fluid concentration at free stream |  | $C_{f}$ |  | Skin friction coefficient |
| $D_{B}$ |  | Brownian diffusion coefficient$\left(m^{2}/s\right)$ |  | $Nu_{x}$ |  | Local Nusselt number |
| $D_{T}$ |  | Thermophoresis diffusion coefficient$\left(m^{2}/s\right)$ |  | $Sh_{x}$ |  | Local Sherwood number |
| $N_{b}$ |  | Brownian diffusion parameter |  | $k$ |  | Thermal conductivity$\left(W/k.m\right)$ |
| $N_{t}$ |  | Thermophoretic parameter |  |  |  | Volume fractions of solid nanoparticles |
| $\Pr$ |  | Prandtl number |  | $\theta$ |  | Dimensionless temperature |
|  |  | Dimensionless concentration |  | $\varepsilon$ |  | Velocity ratio parameter |
| $\rho_{f}$ |  | Density of fluid$\left(kg/m^{3}\right)$ |  | $\mu$ |  | Dynamic viscosity$\left(kg/m^{2}s\right)$ |
| $\upsilon_{f}$ |  | Kinematic viscosity$\left(m^{2}/s\right)$ |  | $\mu_{hnf}$ |  | Dynamic viscosity of hybrid nanofluid $\left(kg/m^{2}s\right)$ |
| $\kappa_{hnf}$ |  | Thermal conductivity of hybrid nanofluid |  | $\rho_{hnf}$ |  | Density of hybrid nanofluid $\left(kg/m^{3}\right)$ |
